# Case Report: Minimally Invasive Therapy by Transcatheter Aortic Valve Replacement and Percutaneous Intramyocardial Septal Radiofrequency Ablation for a Patient With Aortic Stenosis Combined With Hypertrophic Obstructive Cardiomyopathy: Two-Year Follow-Up Results

**DOI:** 10.3389/fcvm.2021.735219

**Published:** 2021-09-20

**Authors:** Yijian Li, Yuan Feng, Xi Li, Lei Zuo, Tao Gu, Liwen Liu, Mao Chen

**Affiliations:** ^1^Department of Cardiology, West China Hospital of Sichuan University, Chengdu, China; ^2^Department of Ultrasound, Xijing Hypertrophic Cardiomyopathy Center, Xijing Hospital, Fourth Military Medical University, Xi'an, China

**Keywords:** minimally invasive therapy, transcatheter aortic valve replacement, percutaneous intramyocardial septal radiofrequency ablation, aortic valve stenosis, hypertrophic obstructive cardiaomyopathy

## Abstract

With the development of minimally invasive technologies in the medical field, more and more technologies can replace surgical thoracotomy and relieve the pain of disease via minimally invasive methods. We reported a case of aortic valve stenosis combined with left ventricular outflow track obstruction treated by two minimally invasive techniques, transcatheter aortic valve replacement and transthoracic echocardiography–guided percutaneous intramyocardial septal radiofrequency ablation, and followed up for 2 years.

## Background

Surgical aortic valve replacement (SAVR) is recommended as first choice for patients with aortic valve stenosis (AS) combined with septal hypertrophy that needed septal reduction therapies, especially for patients with left ventricular outflow track obstruction (LVOTO) (1). With the development of minimally invasive therapy, transcatheter aortic valve replacement (TAVR) has been recommended for symptomatic aortic stenosis (AS) in patients of all risk categories (2). Nevertheless, TAVR has not been used in patients with aortic stenosis combined with LVOTO. For these patients, possibly minimally invasive therapy is septal alcohol ablation after TAVR. Although alcohol septal ablation is recommended for patients with LVOTO, it is associated with high risk of complications, arrhythmic events, and poor long-term prognosis. Recently, a novel minimally invasive treatment, transthoracic echocardiography (TTE)–guided percutaneous intramyocardial septal radiofrequency ablation (PIMSRA), has been reported in small numbers of hypertrophic cardiomyopathy patients (3). Thus, PIMSRA can be used for patients with aortic valve stenosis combined with LVOTO as a novel, less invasive therapy.

## Case Presentation

A 62-year-old man complained of dyspnea and amaurosis for 2 years. For ~2 years before admission, the patient had transient tightness in the chest and dyspnea on exertion. Coronary angiography was performed at the local hospital, suggesting mild stenosis of the right coronary artery. After that, he was prescribed with aspirin 100 mg and atorvastatin 10 mg, and his symptoms did not improve significantly. Two months ago, he experienced an exacerbation of his dyspnea accompanied by amaurosis, which arose during minimal physical activity. Therefore, he was admitted to our hospital with New York Heart Association (NYHA) functional class III. Physical examination was unremarkable, and vital signs were normal. The patient did not measure his blood pressure (BP) regularly and his BP measured during hospitalization was 110–120/70–80 mmHg. His resting heart rate was 70–80 beats per minute.

**Figure 1:**
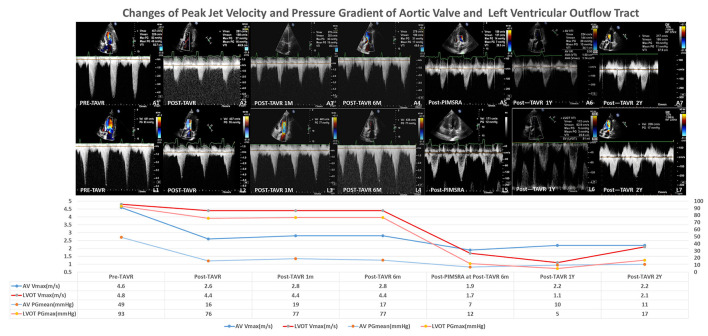
Changes of peak jet velocity and pressure gradient of aortic valve and left ventricular outflow track.

**Figure 2:**
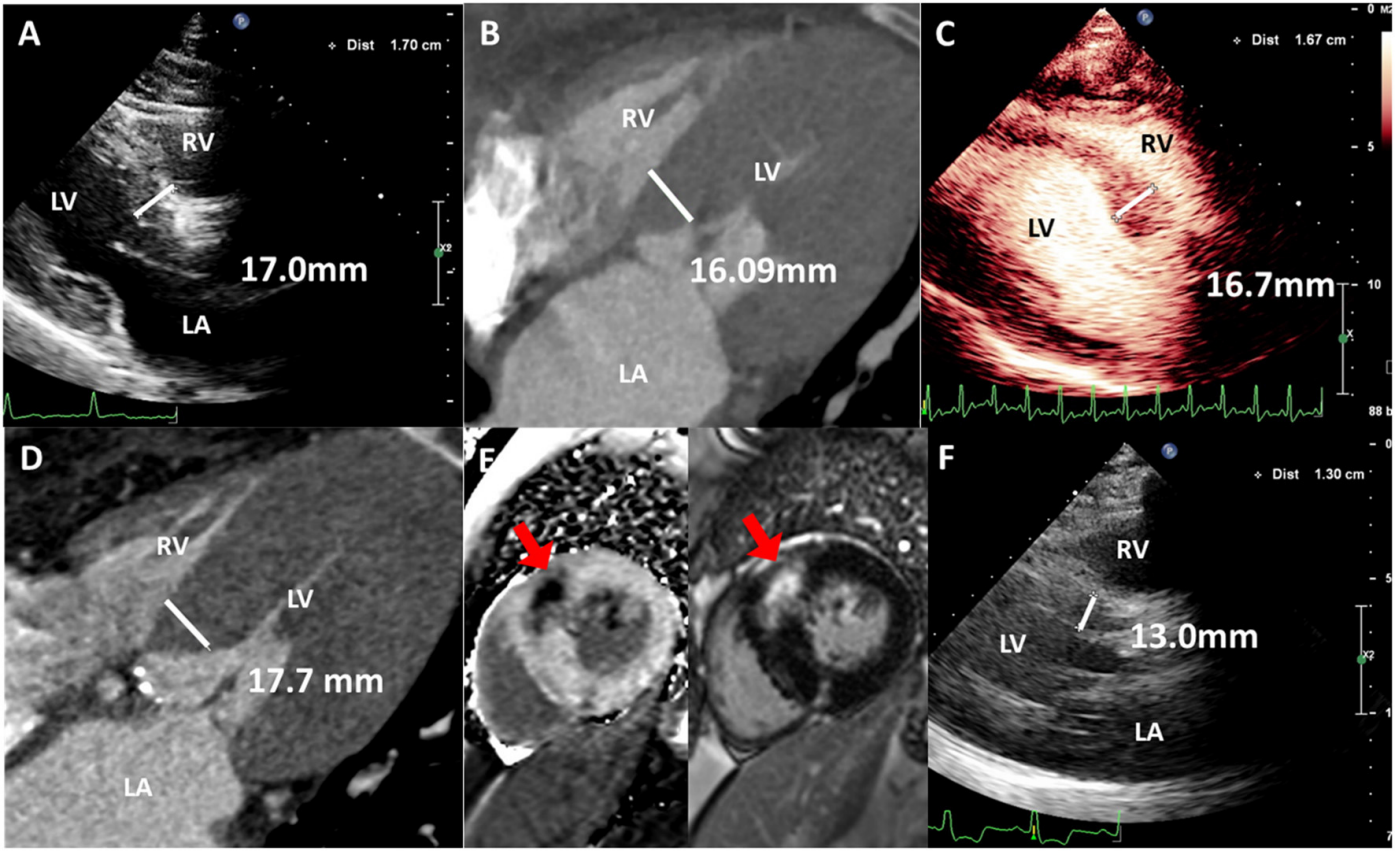
Imaging characteristics of interventricular septa. **(A)** Thickened interventricular septa was confirmed by TTE of 17.0 mm. **(B)** Thickened interventricular septa was confirmed by CT of 16.09 mm. **(C)** Left ventricular contrast echocardiography shows thickened interventricular septa of 16.7 mm at 6-month follow-up after TAVR. **(D)** Thickened interventricular septa was confirmed by CT of 17.7 mm at 6-month follow-up after TAVR. **(E)** Myocardial fibrosis and delayed enhancement was confirmed by cardiac magnetic resonance. **(F)** Interventricular septal thickness decreased to 13.0 mm after PIMSRA 5 days later. Red arrows: delayed enhancement.

**Figure 3:**
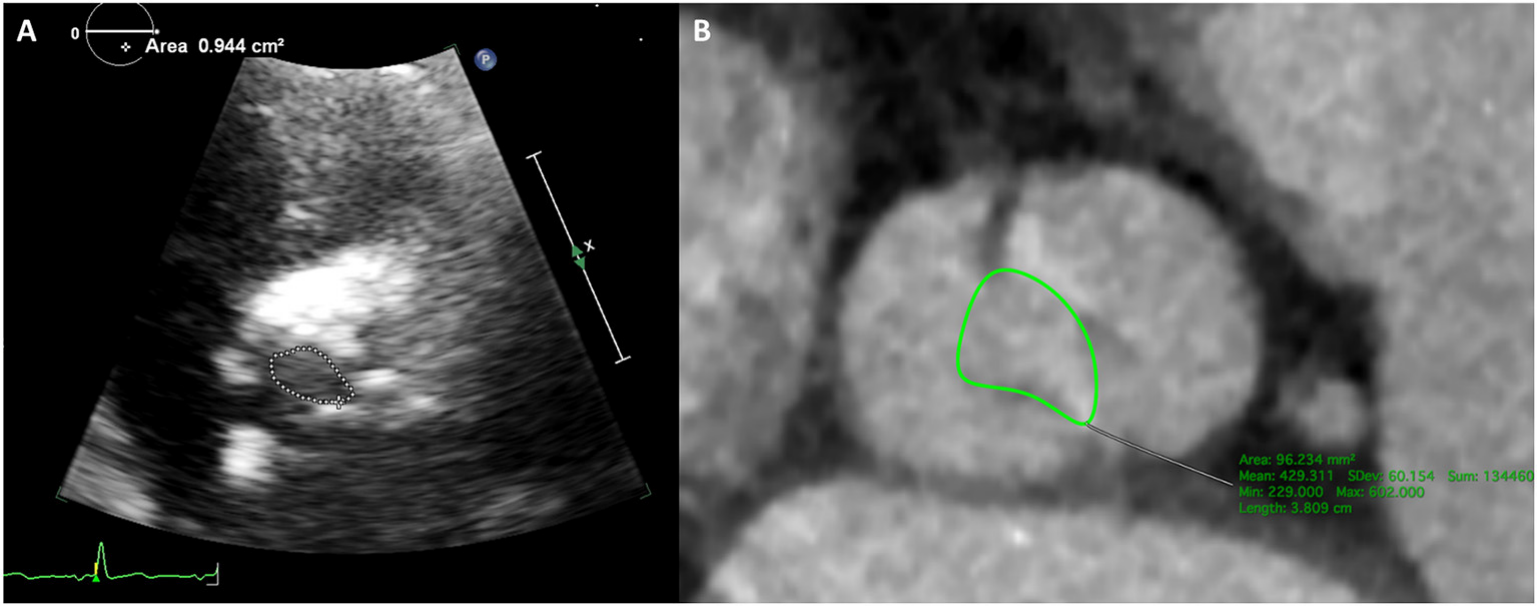
Type-0 bicuspid aortic valve orifice area measured by echocardiography and CT. **(A)** Aortic valve orifice area was 0.94 cm^2^ measured by TTE. **(B)** Aortic valve orifice area was 0.96 cm^2^ measured by CT of systolic phase.

**Figure 4:**
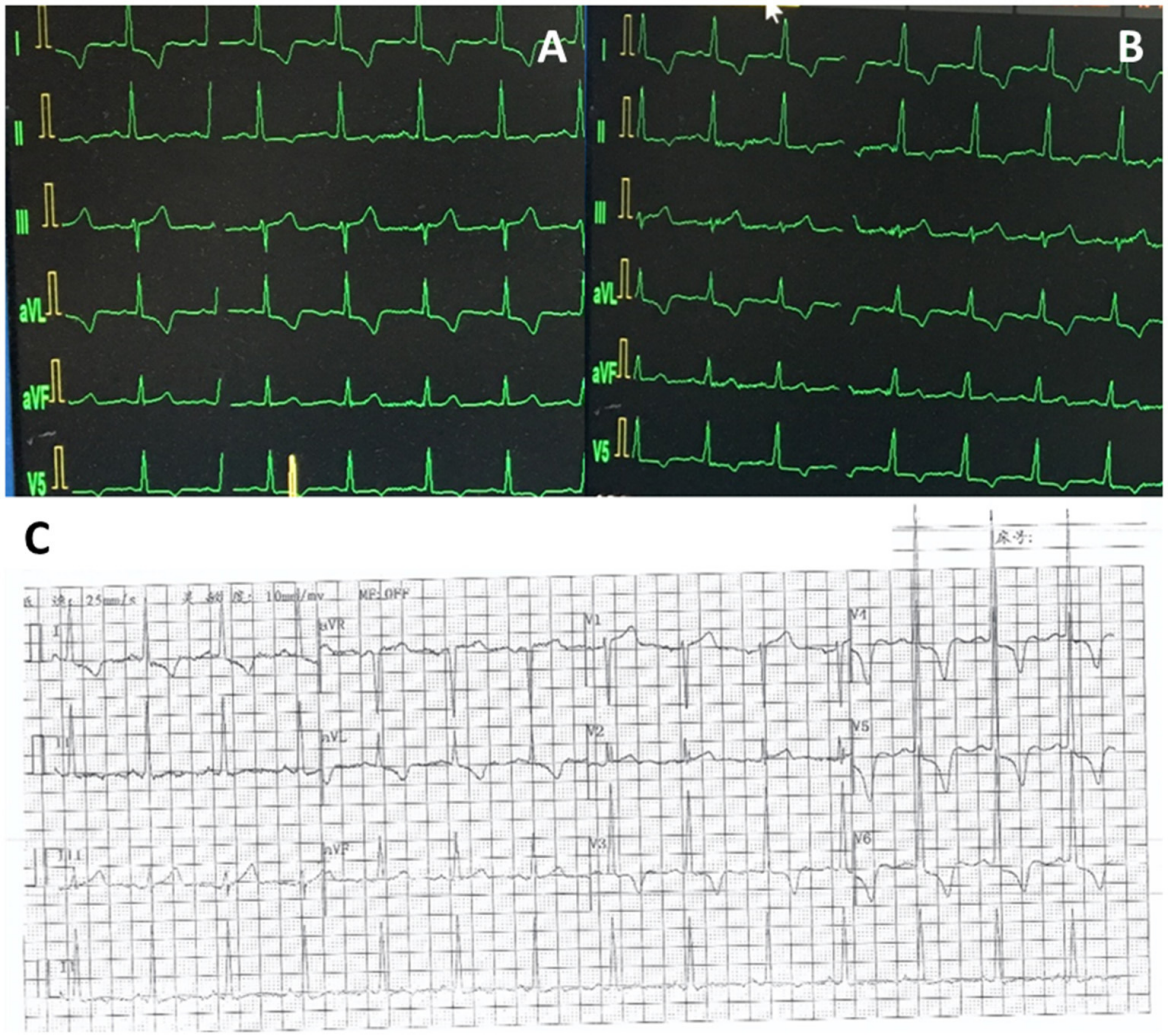
ECGs at different times. **(A)** Pre-TAVR ECG. **(B)** Post-TAVR ECG. **(C)** ECG at 6-month follow-up after TAVR.

TTE suggested severe aortic stenosis (AS) (peak jet velocity 4.6 m/s, mean pressure gradient 49 mmHg) combined with LVOTO (peak jet velocity 4.8 m/s, maximum pressure gradient 93 mmHg) (Figure 1). Interventricular septal thickening (16–17 mm) was confirmed by TTE and CT (Figures 2A,B; Supplementary Videos 1, 2). Although the image collection of TTE was not satisfactory enough, the TTE showed and CT confirmed the diagnosis of type-0 bicuspid calcific aortic stenosis. Aortic valve area was 0.94 cm^2^ measured by echocardiography and was 0.96 cm^2^ measured by CT of systolic phase (Figures 3A,B; Supplementary Videos 3, 4). As the patient was diagnosed with AS and suspected with hypertrophic obstructive cardiomyopathy (HOCM), aortic valve replacement and septal myectomy was suggested by heart team consultation (1, 4). However, the patient declined surgery even with a low surgical risk (Society of Thoracic Surgeons score 1.399%) and demanded for less invasive therapy. Considering the difficulty of alcohol septal ablation (ASA) at the septal branch of this patient, TAVR combined with PIMSRA was planned. As the balloon-expandable valve had not been approved for sale in mainland China at that time, we used the only one domestic self-expandable Venus A-valve (Venus MedTech, Hangzhou, Inc., China) for TAVR. Pre-TAVR six-lead ECG findings included sinus rhythm, inverted T waves at the inferior and lateral leads (I, II, AVL, and V5), and depressed ST segment at leads I and AVL (Figure 4A). After a 26-mm self-expandable Venus A-valve was successfully deployed, the post-TAVR mean pressure gradient decreased to 17 mmHg and peak jet velocity to 2.8 m/s, but LVOTO remained unchanged (Figure 1, Supplementary Videos 5, 6). There was no significant change observed by post-TAVR six-lead ECG without any complications of conduction block (Figure 4B). Because his BP was not stable when 5 mg bisoprolol fumarate was added, we maintained the maximum dose he could take. He was prescribed with clopidogrel 75 mg, aspirin 100 mg, bisoprolol fumarate 2.5 mg, atorvastatin 20 mg, furosemide 20 mg, spirolactone 20 mg, and potassium chloride 1 g at discharge with NYHA functional class II. At 6 months' follow-up after TAVR, he complained of dyspnea again with NYHA functional class III. There were no significant changes in the follow-up 12-lead ECG (Figure 4C). LVOTO (peak jet velocity 4.4 m/s and maximum pressure gradient 77 mmHg) (Figure 1) and septal thickening were still shown by TTE and CT; delayed enhancement of myocardium was confirmed by cardiac magnetic resonance imaging (CMR) (Figures 2C–E). Although the gene detection showed that no HOCM-related genes were found, the diagnosis of HOCM has been proved. Although ASA is recommended for patients with HOCM, it is associated with high risk of conduction block and high recurrence rate (5). Thus, PIMSRA became a possible choice (3). The maximum pressure gradient of LVOT decreased to 12 mmHg after PIMSRA immediately (Figure 2F; Supplementary Videos 7, 8), and the thickness of the septa decreased to 13 mm 5 days later. The mean pressure of the aortic valve reached 7 mmHg (Figure 1). The patient's symptoms were completely relieved at discharge with NYHA functional class II. He was prescribed aspirin 100 mg and metoprolol succinate 47.5 mg. After follow-up at 2 years after TAVR, 1.5 years after PIMSRA, the patient's symptoms remained stable, and echocardiography showed peak jet velocity of aortic valve, peak jet velocity of LVOT, and thickness of septa of 2.5 m/s, 2.1 m/s, and 13 mm, respectively (Figure 1; Supplementary Video 9).

## Discussion and Conclusion

**Figure 5:**
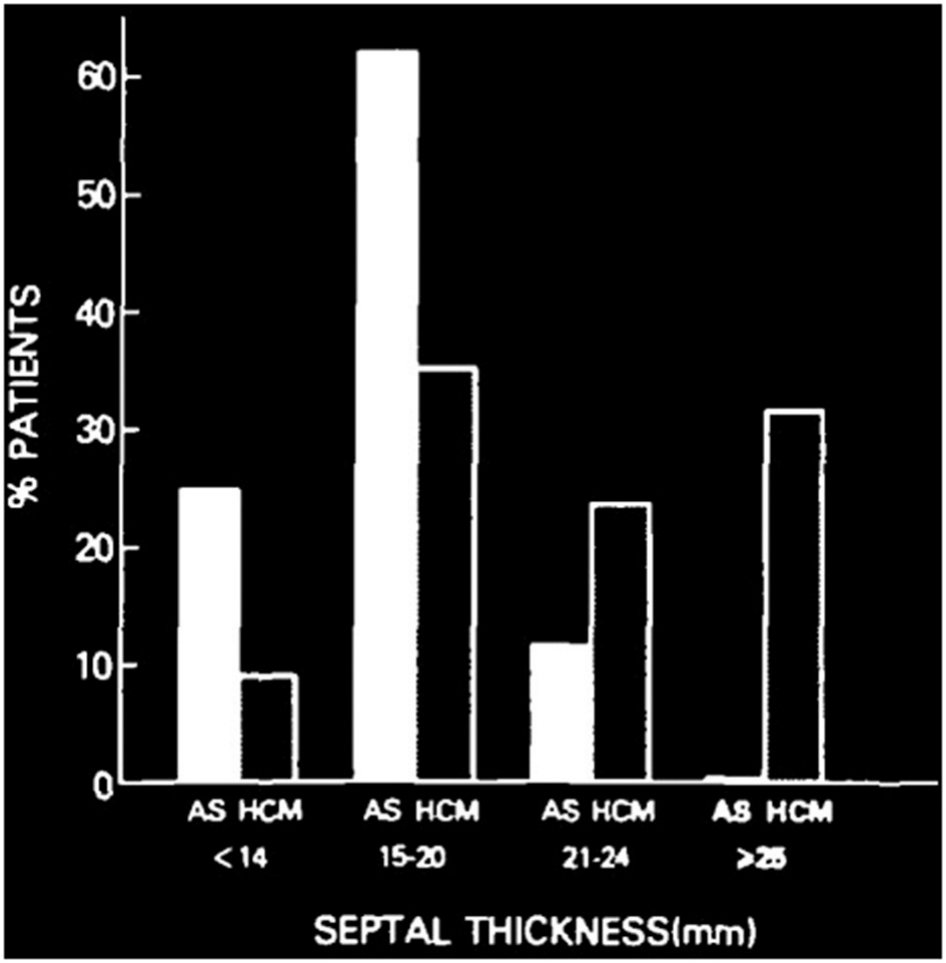
Difference of ventricular septal thickness between valvular aortic stenosis and hypertrophic cardiomyopathy. Comparison of ventricular septal thickness in 85 patients with valvular aortic stenosis (AS) and 151 patients with hypertrophic cardiomyopathy (HCM), studied with echocardiography and at necropsy (5).

A previous study showed that there was a difference in septal thickness between AS and HOCM. Septal thickness more than 25 mm is uncommon for patients with AS. Also, for patients with septal thickness between 15 and 20 mm, 60% were AS patients and 40% were HOCM (Figure 5) (6). In this case, the septal thickness of 17 mm cannot diagnose AS or HOCM. Although the gene detection showed no HOCM-related genes were found, the asymmetric patterns of left ventricular hypertrophy, the LVOTO with maximum pressure gradient 93 mmHg, and delayed enhancement of myocardium by CMR, all the evidences support the diagnosis of HOCM. Based on existing clinical characteristics, SAVR is the first choice for patient concomitant with AS and LVOTO (1, 4). With the development of minimally invasive therapy in the cardiovascular field, more and more patients are beginning to request the use of minimally invasive technology to relieve their symptoms. As a less invasive therapy for obstructive HCOM, ASA has been proven as a safe and effective therapy for LVOTO, about nearly 80% success rate after ASA compared with more than 90% success rate after myectomy. Previous studies showed short-term results that TAVR and ASA were safe and effective for concomitant AS and obstructive HCOM (7). However, several disadvantages of ASA should be mentioned (5, 8). Nearly 10% of ASA patients require a permanent pacemaker and more than 7% of ASA patients required reintervention (8). As a novel therapy, PIMSRA has showed advantages in success rate and complication rates. This is a percutaneous intramyocardial, non-transaortic, and non-transcoronary approach, which could reduce LVOTO and avoid sternotomy, rely on alcohol injection, and damage the conduction system distributed underneath the endocardium. In this case, TAVR as a less invasive technique was used for AS treatment. However, after TAVR 6-month follow-up, the patient has relapsed symptoms caused by LVOTO. Considering the high risk of complication and high recurrence rate of ASA and the difficulty to get the coronary artery after TAVR, PIMSRA became another choice. A previous study (9) showed the concept of suicide left ventricule (LV) from a case that was diagnosed with AS, small LV, thickened septa, and hyperdynamic LV systolic function. After TAVR procedure, the patient's acute hemodynamic derangement was secondary to HOCM physiology although there was absence of systolic anterior motion (SAM) of the anterior mitral valve leaflet before TAVR. In our case, the patient did not experience acute LV function changes after TAVR, but the patient's symptoms relapsed after TAVR 6 months caused by LVOTO, which required a second procedure. Under the guarantee of a multi-disciplinary heart team, simultaneous TAVR and PIMSRA may be a better therapy for these patients. At 2-year follow-up, TAVR and PIMSRA showed safety and efficacy to treat concomitant AS and obstructive HOCM. For patients with AS combined with obstructive HOCM, TAVR combined with PIMSRA is a novel minimally invasive therapy. Besides, this technique may offer new insight for patients with residual pressure after TAVR caused by secondary LVOTO.

## Data Availability Statement

The original contributions generated for the study are included in the article/Supplementary Material, further inquiries can be directed to the corresponding author/s.

## Ethics Statement

The study protocol was approved by the Ethics Committee of the West China Hospital, Sichuan, China. The patients/participants provided their written informed consent to participate in this study. Written informed consent was obtained from the individual(s) for the publication of any potentially identifiable images or data included in this article.

## Author Contributions

YL drafted the article. MC and LL designed the study. YF, XL, TG, and LZ revised the article. YL, XL, TG, and LZ were responsible for the collection of data or analysis. All authors read and approved the final article.

## Funding

This work was supported by the 1.3.5 Project for Disciplines of Excellence, West China Hospital, Sichuan University; China Postdoctoral Science Foundation (229304).

## Conflict of Interest

MC and YF are consultants/proctors of Venus MedTech. The remaining authors declare that the research was conducted in the absence of any commercial or financial relationships that could be construed as a potential conflict of interest.

## Publisher's Note

All claims expressed in this article are solely those of the authors and do not necessarily represent those of their affiliated organizations, or those of the publisher, the editors and the reviewers. Any product that may be evaluated in this article, or claim that may be made by its manufacturer, is not guaranteed or endorsed by the publisher.

## Supplementary Material

Supplementary Video 1 Thickened interventricular septa was confirmed by TTE (left ventricular long-axis view).

Supplementary Video 2 Thickened interventricular septa was confirmed by TTE (four-chamber view).

Supplementary Video 3 Short-axis view of type-0 bicuspid aortic valve stenosis by echocardiography.

Supplementary Video 4 En face view of type-0 bicuspid aortic valve stenosis by CT.

Supplementary Video 5 Successful TAVR procedure without any adverse events is shown by angiography.

Supplementary Video 6 LVOTO is shown by left ventricular angiography.

Supplementary Video 7 Radiofrequency electrode needle was inserted into the hypertrophied IVS via the percutaneous intramyocardial approach.

Supplementary Video 8 The thickness of interventricular septa was significantly reduced after PIMSRA for 5 days (four-chamber view by TTE).

Supplementary Video 9 Follow-up at 1.5 years after PIMSRA and 2 years after TAVR. The thickness of interventricular septa was maintained at 13 mm (four-chamber view by TTE).
